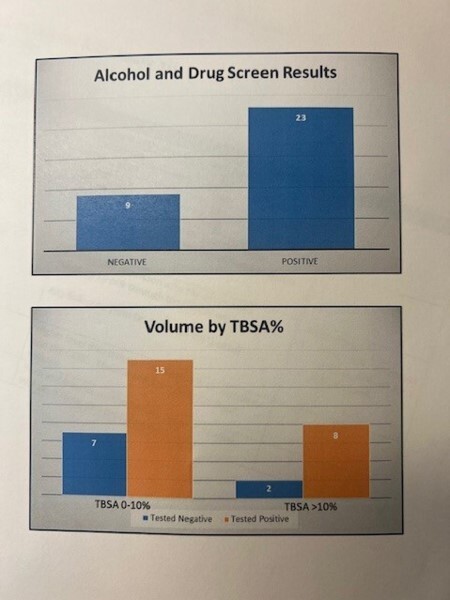# 729 The Impact of Alcohol Use on Recreational Burn Admissions from Campfires and Bonfires

**DOI:** 10.1093/jbcr/irae036.272

**Published:** 2024-04-17

**Authors:** Heidi M Altamirano, Mark J Johnston, Sam A Miotke

**Affiliations:** Regions Hospital, St. Paul, MN; Regions Hospital, St. Paul, MN; Regions Hospital, St. Paul, MN

## Abstract

**Introduction:**

According to the American Burn Association, the incidence of burn injuries requiring medical treatment is over 486,000 per year. Of these, 40,000 require hospitalization for their injuries, and 5% are related to recreational burns. The increasing trend of admissions due to recreational fires along with the incidence of trauma and burn related admissions association with alcohol or drug use prompted this review.

**Methods:**

A retrospective chart review of admissions due to burn injury from campfires or bonfires treated at a single center was conducted. Admitted patients of all ages from January 2018 through July 2023 were included. Charts were reviewed, and data abstraction included demographic information, epidemiologic data and clinical characteristics. The associated use of alcohol or drugs was identified to create comparison groups (tested positive or negative) to analyze the TBSA burned, length of stay, use of accelerants, and other factors.

**Results:**

Ninety-nine patients were admitted to the burn center with injuries related to campfire or bonfires during this timeframe. Seventy percent were adult patients while 30% were children under the age of 17. Eighty-one percent of all patients were under the age of 40. Patients used accelerants in the campfire or bonfire in 30% of cases. Eighty-one percent had TBSA burns less than 10%.

Thirty percent (32) of patients had alcohol or drug screens completed on admission. Of these, 72% (23) tested positive for alcohol or drugs. Fifty-six percent were males, while 44% were females. The group testing positive had longer hospital lengths of stay compared to those testing negative, 10 days compared to 4. Those testing positive had more surgical procedures than those testing negative, 62% versus 33%. The use of accelerants in the campfire or bonfire by patients testing positive versus negative were 43% compared to 33%. Overall, the group testing positive had a total of 43 surgical procedures and 506 days in the hospital, while the group testing negative had 6 surgeries and 98 hospitalized days.

**Conclusions:**

The correlation between use of alcohol or drugs and injury due to trauma and burn is well known. The American College of Surgeons Committee on Trauma requires for verification that all injured patients must be screened for alcohol use and if screened positive, that an intervention be performed. This study looked at the impact on a specific subset of burn injured patients from campfires and bonfires. The group testing positive for alcohol or drug use had larger burn injuries, required more surgical procedures, and longer hospital lengths of stay. The need for ongoing education, prevention, and advocacy is needed.

**Applicability of Research to Practice:**

The impact of these injuries to the overall health care system is significant. The impact of these life-long injuries for patients and their families is also significant. This research is applicable to practice as it can be used to strengthen the support for education, prevention, and advocacy.